# Progress in research on predictors of adverse outcomes in patients with nasal inflammatory diseases

**DOI:** 10.3389/fimmu.2025.1740005

**Published:** 2026-01-15

**Authors:** Jiang-Xue Liao, Xin Lin, Jing He, Hua-Jun Feng, Zhuo-Ping Liang, Gang Qin

**Affiliations:** Department of Otolaryngology Head and Neck Surgery, The Affiliated Hospital of Southwest Medical University, Luzhou, China

**Keywords:** allergic rhinitis, chronic rhinosinusitis, nasal inflammatory diseases, precision medicine, predictive markers

## Abstract

Nasal inflammatory disease has a complex pathogenesis, high incidence and long disease course. Complete resolution is often challenging, and these diseases are closely related to upper and lower respiratory tract diseases. For common nasal inflammatory diseases, such as chronic rhinosinusitis (CRS), allergic rhinitis (AR), and fungal rhinosinusitis (FRS), adverse outcomes, such as repeated inflammation, AR combined with asthma, and postoperative recurrence, often occur despite standardized treatments, causing great distress to patients and increasing societal costs due to the need for long-term and repeated treatments. Therefore, the identification of early predictors of unfavorable outcomes of nasal inflammatory diseases is important for achieving early diagnosis, intervention and treatment of nasal inflammatory diseases. This paper summarizes the progress in research on the role of indicators, such as inflammatory cytokines, inflammatory cells, metabolites, nasal flora, and clinical parameters, in predicting poor outcomes in patients with nasal inflammatory diseases.

## Graphical Abstract

**Figure:**
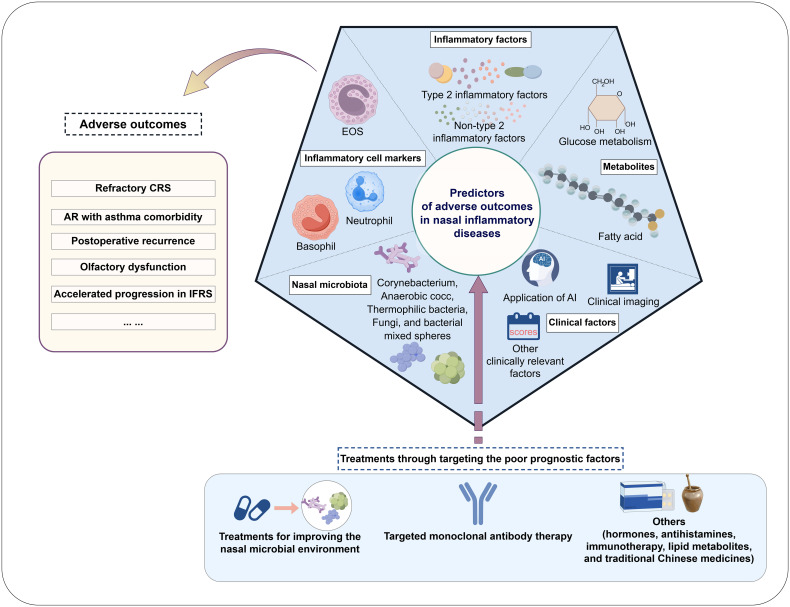
Schematic of predictors of adverse outcomes and targeted interventions in nasal inflammatory diseases. This graphical abstract outlines key poor outcomes (e.g., refractory CRS, AR with asthma comorbidity, postoperative recurrence, olfactory dysfunction, accelerated progression in lFRs), core predictive indicators (inflammatory factors, inflammatory cell markers, metabolites, nasal microbiota and clinical factors), and corresponding targeted interventions (e.g., monoclonal antibody therapy, nasal microbial modulation) in nasal inflammatory diseases. EOS, eosinophil; AI, artificial intelligence; CRS, chronic rhinosinusitis; AR, allergic rhinitis; IFRS, invasive fungal rhinosinusitis.

## 1 Introduction

Nasal inflammatory diseases encompass acute and chronic conditions in the nasal cavity and sinus mucosa caused by allergic, infectious and other pathological factors and autoimmune problems. These diseases include mainly chronic rhinosinusitis (CRS), allergic rhinitis (AR), and fungal rhinosinusitis (FRS). With changes in lifestyle, the acceleration of industrialization, and the extensive use of antibiotics and glucocorticoids, the incidence of nasal inflammatory diseases is gradually increasing. According to a study from seven centers in China, the overall prevalence of CRS is 8.0% (4.8%-9.7%) (1). The prevalence of AR in adults is approximately 17% to 28.5% in Europe and 10% to 30% in the United States (2). From 2005–2011, the prevalence of AR among Chinese adults increased from 11.1% to 17.6% (3). FRS is the cause of approximately 10% of all sinus surgeries, and the incidence of fungal maxillary rhinosinusitis is 15%-20% (4).

A cross-sectional observational study on the burden of respiratory diseases in the Asia–Pacific region revealed that the cost of each patient with respiratory diseases (including CRS, AR, asthma, and chronic obstructive pulmonary disease) is approximately $1,495 per year, and CRS and AR are major causes of decreased patient productivity (5). Despite standardized treatments involving drugs or drugs combined with surgery, disease control in nearly 30% of patients with chronic rhinosinusitis with nasal polyps (CRSwNP) is still poor, with some patients experiencing disease recurrence (6).

AR affects approximately 80% of asthma patients and is a risk factor for the development and exacerbation of asthma (7). Furthermore, if fungal maxillary rhinosinusitis is not effectively treated and cured, there is a risk of recurrence.

Therefore, nasal inflammatory diseases have become serious global public health problems. Actively identifying early indicators of the unfavorable prognosis of nasal inflammatory diseases and providing standardized clinical intervention as soon as possible are critical for improving the quality of life (QoL) and outcomes of patients and reducing societal and economic costs. In developing countries, the efficiency of hospital admission and the appropriateness of hospitalization often remain overlooked, despite being key factors in health system performance. It is essential to utilize various markers of hospital performance and patient characteristics to assess the appropriateness of hospitalization. This aligns closely with identifying predictive factors in chronic diseases, where early recognition of adverse outcomes is crucial for patient care and resource allocation (8).

Although numerous high-quality reviews have elucidated the pathophysiology of nasal inflammatory diseases and the potential adverse prognostic indicators implicated, these works tend to be constrained by narrow scopes: For example, they often focus on a single disease subtype (e.g., exclusively AR or CRS) (9, 10), a single research dimension (e.g., solely the nasal microbiome or biomarkers) (11, 12), a limited subset of biomarkers (e.g., one category or individual biomarker) (13–15), or only current biological agents (16). In contrast, this review synthesizes the latest research on factors predicting a poor prognosis in patients with a range of clinically prevalent nasal inflammatory diseases, including CRS, AR, and FRS; moreover, this review integrates multidimensional predictive indicators, such as inflammatory factors, inflammatory cells, metabolomic markers, nasal microbiome characteristics, radiomic features, and machine learning-driven artificial intelligence (AI) integration models. Furthermore, this review summarizes the clinical application status of these indicators, aiming to provide a more holistic overview of these diseases and lay a theoretical foundation for developing a clinically feasible early warning system. Overall, the goal of this review is to facilitate the transition of nasal inflammatory disease management from a passive responsive model to one centered on proactive prediction and personalized prevention.

## 2 Literature search and selection methods

### 2.1 Literature search strategy

To guarantee a comprehensive and systematic retrieval of relevant literature, a structured search was conducted in strict adherence to the Preferred Reporting Items for Systematic Reviews and Meta-Analyses (PRISMA) 2020 Statement. Four core electronic databases—PubMed, Embase, the Web of Science Core Collection, and the Cochrane Library—were queried for literature published between January 2000 and June 2025. The search strategy integrated both Medical Subject Headings (MeSH) terms and free-text keywords pertaining to the core research themes: “nasal inflammatory diseases”, “chronic rhinosinusitis”, “allergic rhinitis”, “predictive markers”, “biomarker”, “artificial intelligence”, and “precision medicine”. Furthermore, the reference lists of all included primary studies and relevant review articles were subjected to manual screening to retrieve additional eligible publications.

### 2.2 Inclusion and exclusion criteria

Studies were selected on the basis of the following predefined criteria:

The inclusion criteria were as follows (1): Original research articles or systematic reviews published in peer-reviewed journals focused on biomarkers or AI applications in nasal inflammatory diseases (e.g., CRS, AR) (2); studies providing explicit disease diagnostic criteria (e.g., the European Position Paper on Rhinosinusitis and Nasal Polyps 2024 [EPOS 2024] for CRS) and quantitative data on biomarker performance (e.g., sensitivity, specificity, area under the curve (AUC)) or AI model efficacy (e.g., accuracy, predictive value) (3); human-subject studies, with a sample size of ≥30 for original research or ≥10 included primary studies for systematic reviews (4); studies aligned with the core research focus of predictive markers and precision medicine in nasal inflammatory diseases; and (5) publications in the English language.

The exclusion criteria were as follows (1): conference abstracts, editorials, case reports (sample<10), and narrative reviews (2); studies conducted solely on animal models or cell lines (no human data) (3); articles with unavailable full texts or unextractable key data (e.g., biomarker AUC values, AI model validation metrics) (4); identical or duplicate publications or studies irrelevant to the core research question; and (5) non-English publications.

### 2.3 Study selection process

The study selection process strictly followed the PRISMA 2020 guidelines. All the retrieved records were imported into EndNote (reference management software) for deduplication. The selection was performed in two stages: First, two authors (Jiang-Xue Liao and Xin Lin) independently screened the titles and abstracts of all the records to meet the inclusion/exclusion criteria. Second, these two authors independently retrieved and evaluated the full texts of potentially eligible studies. Any discrepancies at either stage were resolved through discussion or, if necessary, by consultation with a third author (Jing He).

### 2.4 Data extraction and synthesis

To ensure consistency, a standardized data extraction form was developed. For each included study, the following information was extracted: first author, publication year, country, study design, sample size, participant characteristics (e.g., CRS subtype, AR, FRS), key methods (e.g., biomarker detection techniques, AI model algorithms), main findings (e.g., the prediction performance and predictive value of biomarkers and AI models), and conclusions. The data were extracted independently by two authors to ensure accuracy. Given the anticipated methodological and clinical heterogeneity among the included studies (e.g., diverse CRS endotypic subtypes, varied AI model architectures), a narrative synthesis approach was adopted. The findings were thematically organized (e.g., “inflammatory factors”, “inflammatory cell markers”, “metabolites”) and presented in subsequent sections, incorporating cross-study comparisons and contrasts to highlight consistencies, discrepancies, and underlying factors driving divergent results.

## 3 Inflammatory factors

### 3.1 Type 2 inflammatory factors

**Figure 1:**
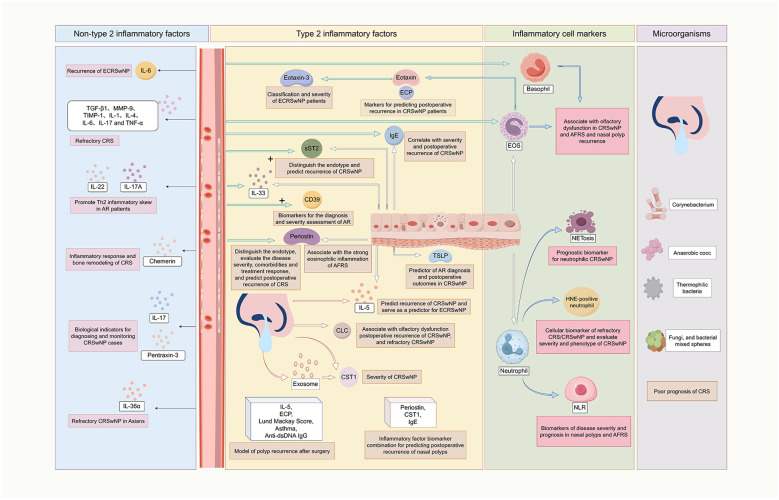
Role of type 2 and non-type 2 inflammatory factors, inflammatory cell markers, and microorganisms in poor outcomes of nasal inflammatory diseases (1). Type 2 inflammatory factors: the expression of IgE in tissues or blood is associated with the severity and postoperative recurrence of CRSwNP. Elevated levels of IL-5 in tissues or nasal secretions can predict postoperative recurrence of CRSwNP and identify ECRSwNP, whereas CLC is associated with olfactory dysfunction, recurrence of CRSwNP, and refractory CRSwNP. A combined model consisting of the IL-5 level, ECP level, preoperative Lund–Mackay score, asthma status, and anti-dsDNA IgG level has good ability to predict postoperative recurrence of nasal polyps. The levels of IL-33 and sST2 in nasal polyp tissues and serum are objective biomarkers for distinguishing the endotype and predicting the recurrence of CRSwNP. Serum CD39 and IL-33 levels may serve as biomarkers for diagnosing AR and indicating its severity. TSLP can be used as a predictive factor for AR diagnosis and postoperative outcomes in CRSwNP patients. Serum periostin can be used to not only differentiate the endotype of CRS patients but also evaluate disease severity, comorbidities, treatment response, and predict postoperative recurrence of CRS. Increased periostin levels in AFRS tissues are associated with strong eosinophilic inflammation. CST1 in exosomes and nasal secretions is related to CRSwNP disease severity. Mucin and serum periostin and mucin CST1 and IgE are the best inflammatory factor biomarker combinations for predicting postoperative recurrence of nasal polyps. The markers ECP and eotaxin for EOS are indicators for predicting postoperative recurrence in CRSwNP patients. Eotaxin-3 levels may also be important markers for the classification and severity of ECRSwNP patients (2). Non-type 2 inflammatory factors: Serum IL-6 levels are related to postoperative recurrence of ECRSwNP. TGF-β1, MMP-9, TIMP-1, IL-1, IL-4, IL-6, IL-17, and TNF-α influence refractory CRS. IL-22 and IL-17A can promote Th2 cell inflammatory skewing in AR patients. Chemerin is related to the inflammatory response and bone remodeling in the context of CRS. IL-17 and pentraxin-3 levels are important biological indicators for diagnosing and monitoring CRSwNP cases. IL-36α is a biomarker for predicting refractory CRSwNP in Asians (3). Inflammatory cell markers: Elevated levels of EOSs/basophils in tissue or blood are significantly related to the recurrence and olfactory dysfunction of CRSwNP. Blood EOS and basophil levels are related to AFRS recurrence. The number of human subepithelial HNE-positive neutrophils can serve as a cell marker for refractory CRS/CRSwNP and is associated with the disease severity and phenotype of CRSwNP. NETosis can serve as a prognostic biomarker for neutrophilic CRSwNP. The NLR can be a marker for the severity and prognosis of nasal polyp and AFRS diseases (4). Microorganisms such as Corynebacterium, anaerobic cocc, thermophilic bacteria, fungi, and bacterial mixed spheres may be indicators of poor prognosis in CRS patients. IgE, immunoglobulin E; CRSwNP, chronic rhinosinusitis with nasal polyps; IL, interleukin; ECRSwNP, eosinophilic chronic rhinosinusitis with nasal polyps; CLC, Charcot-Leyden crystal; ECP, eosinophil cationic protein; anti-dsDNA, anti-double-stranded DNA; sST2, soluble ST2; AR, allergic rhinitis; TSLP, thymic stromal lymphopoietin; CRS, chronic rhinosinusitis; AFRS, allergic fungal rhinosinusitis; CST1, Cystatin C1; EOS, eosinophil; TGF-β1, transforming growth factor-β1; MMP-9, matrix metalloproteinase 9; TIMP-1, tissue inhibitor of metalloproteinase 1; TNF-α, tumor necrosis factor-α; Th2, T helper 2; HNE, human neutrophil elastase; NETosis, neutrophil extracellular trap formation; NLR, neutrophil-lymphocyte ratio.

CRSwNP, a refractory subtype of CRS, is closely related to type 2 inflammation and has a poor prognosis, including poor disease control and recurrence. Analysis of the biomarker profiles of blood and nasal secretion samples from CRSwNP patients before and after surgery revealed that the expression profile of type 2 inflammatory cytokines may be related to CRSwNP-related postoperative recurrence and can predict the efficacy of optimal or targeted drugs and surgical treatment (17). Some studies have suggested that immunoglobulin E (IgE) expression in tissues is correlated with disease severity, clinical and pathological features, and the speed of postoperative recurrence of CRSwNP and that the peripheral blood specific immunoglobulin E (sIgE) expression level (AUC = 0.786) can be used as a reliable indicator of postoperative recurrence of CRSwNP (18) (**Table 1**). Numerous studies have shown that increased IL-5 levels in nasal secretions or polyp tissues are significantly positively correlated with the risk of uncontrolled disease conditions (19) and recurrence (20) of CRSwNP; furthermore, the IL-5 level is a strong predictor for the early diagnosis of eosinophilic chronic rhinosinusitis with nasal polyps (ECRSwNP) (21). Bai et al. (22) reported that the levels of IL-5 and eosinophilic cationic protein (ECP), the preoperative Lund–Mackay score, and the combination of asthma and anti-double-stranded DNA (anti-dsDNA) IgG have a good ability to predict polyp recurrence after endoscopic surgery (AUC = 0.89) (**Table 1**). Several studies have shown that IL-33 and soluble ST2 (sST2) levels in the nasal polyp tissues and serum of CRSwNP patients are correlated with mucosal eosinophil (EOS) infiltration and postoperative recurrence, and these levels might be objective biomarkers for the differentiation of the CRSwNP endotype and the prediction of recurrence (19, 23). Jiang et al. (24) reported that serum CD39 and IL-33 levels, whose cutoff values were 125.9 pg/ml and 242.3 pg/ml, respectively, had a strong diagnostic ability for AR patients, and the receiver operating characteristic (ROC) curves suggested that CD39 can also be used to distinguish AR cases on the basis of severity (**Table 1**). Thymic stromal lymphopoietin (TSLP), a master regulator of type 2 immune responses and atopic diseases, can be used as a predictive factor for atopic diseases such as AR (25). One study indicated that the level of TSLP in the nasal mucosal tissue of CRSwNP patients was associated with a greater improvement in the SNOT-22 score after functional endoscopic sinus surgery, suggesting that the TSLP level can serve as a predictor of postoperative outcomes in CRSwNP patients (26). Another recent study indicated that a baseline plasma TSLP concentration >330 fg/mL can serve as a predictive biomarker for the efficacy of targeted therapy in CRSwNP patients (27) (**Figure 1**) (**Table 1**).

**Table 1:** Key predictive markers of nasal inflammatory diseases.

Predictor		Sample	Disease	Cutoff value	Results	References
Type 2 inflammatory factors	sIgE	Blood	CRSwNP	Youden index =0.595	Predicts the postoperative recurrence of CRSwNP	(18)
	IL-5+ECP+Lund Mackay score+asthma+anti-dsDNA IgG	Tissue	CRSwNP	AUC=0.89	Predicts CRSwNP recurrence	(22)
	IL-33	Serum	CRSwNP	98.9 pg/ml	Forecasts CRSwNP endotypes	(23)
				118.7 pg/ml	Forecasts the recurrence of CRSwNP post-surgery	
			AR	242.3 pg/ml	Early diagnosis of AR	(24)
	sST2	Serum	CRSwNP	25.1 ng/ml	Forecasts CRSwNP endotypes	(23)
				32.1 ng/ml	Forecasts the recurrence of CRSwNP post-surgery	
	TSLP	Plasma	CRSwNP	330 fg/mL	Predicts the treatment efficacy of CM326 for CRSwNP	(27)
	Periostin	Serum	ECRSwNP	130 ng/ml	Predicts the recurrence of ECRSwNP after surgery	(28)
			CRSwNP	115.5 ng/ml	Predicts the recurrence of CRSwNP post-surgery	(30)
				16.826 ng/ml	Predicts the prognosis of CRSwNP	(31)
			CRS	93.0 ng/ml	Discriminates comorbid asthma among CRS	(29)
		Tissue	CRSwNP	8.995 ng/ml	Predicts the prognosis of CRSwNP	(31)
	CLC	Tissue	CRSwNP	1/HPF (crystalline CLC structure)	Forecasts postoperative CRSwNP recurrence	(36)
		Nasal secretion		34.243 ng/mL	Predicts postoperative CRSwNP recurrence	(37)
	Eotaxin	Serum	CRSwNP	43.6 pg/ml	Predicts CRSwNP recurrence after surgery	(38)
Non-type 2 inflammatory factors	IL-6	Serum	ECRSwNP	Youden index =0.470	Predicts the postoperative recurrence of ECRSwNP	(18)
	CD39	Serum	AR	125.9 pg/ml	Early diagnosis of AR	(24)
				71.2 pg/ml	Predicts the severity of AR	(24)
	IL-17	Serum	CRSwNP	9.2 pg/ml	Diagnosis and follow-up of CRSwNP	(42)
	Pentraxin-3	Serum	CRSwNP	4.12 ng/ml	Diagnosis and follow-up of CRSwNP	(42)
Inflammatory cell markers	EOSs	Tissue	ECRSwNP	·EOS count=48/HPF ·EOS ratio=20.6%	Predicts postoperative ECRSwNP recurrence	(6)
			CRSwNP	·EOS count=55/HPF ·EOS ratio=27%	Predicts postoperative CRSwNP recurrence	(44)
		Blood	ECRSwNP	EOS ratio= 2.5%	Predicts postoperative ECRSwNP recurrence	(6)
			CRSwNP	EOS ratio=3.7%	Predicts CRSwNP recurrence	(45)
	Neutrophils	Tissue	CRSwNP	HNE-positive neutrophil number =45/HPF	Predicts refractory CRSwNP recurrence	(40)
Metabolism-related biomarker	ALOX15	Tissue	ECRSwNP	ALOX15 mRNA level (− ΔCt value) =-2.113	Predicts the diagnosis of ECRSwNP	(55)
	P-gp	Nasal secretion	CRSwNP	250 pcg/μg	Predicts the severity of CRSwNP	(58)
	HbA1c	Blood	AIFRS	HbA1c ratio=9.35%	Predicts the poor prognosis of AIFRS	(61)
Clinical imaging	E/M ratio	CT	CRSwNP	2.55	Predicts the recurrence of CRSwNP	(66)

sIgE, specific immunoglobulin E; CRSwNP, chronic rhinosinusitis with nasal polyps; IL, interleukin; ECP, eosinophilic cationic protein; anti-dsDNA IgG, anti-double-stranded DNA; AUC, area under the curve; AR, allergic rhinitis; sST2, soluble ST2; TSLP, thymic stromal lymphopoietin; ECRSwNP, eosinophilic chronic rhinosinusitis with nasal polyps; CLC, Charcot-Leyden Crystal; CRS, chronic rhinosinusitis; HPF, high-power fields; EOS, eosinophil; HNE, human neutrophil elastase; ALOX15, arachidonate 15-lipoxygenase; P-gp, P-glycoprotein; AIFRS, acute invasive fungal rhinosinusitis; E/M ratio, the threshold ratio of total ethmoid sinus (E) and total maxillary (M) scores; CT, computed tomography.

Serum periostin levels can be used not only in the differentiation of CRS endotypes but also in the evaluation of the severity, comorbidities, prognosis and treatment response, especially in the prediction of recurrence after surgery (28–31) (**Table 1**). Laury et al. (32) reported that the increase in periostin in the tissues of allergic fungal rhinosinusitis (AFRS) patients was related to strong eosinophilic inflammation. Some studies have shown that the concentration of cystatin SN (CST1) in exosomes and nasal secretions is correlated with the severity of CRSwNP and could be used as an indicator of poor outcomes in patients with CRSwNP (33). Mueller et al. (34) reported that mucus and serum levels of periostin, mucus, CST1 and IgE might be the best combinations of inflammatory factors for tracking nasal polyps and predicting recurrence. Charcot–Leyden Crystal (CLC)/Gal-10 is considered a marker for the involvement of EOS in allergic reactions and type 2-related inflammation. Studies have shown that the CLC level in nasal secretions is positively correlated with the EOS percentage in nasal polyp tissues and peripheral blood and is negatively correlated with the olfactory threshold (35), and a crystalline CLC structure higher than 1 per high-power field in tissue or a CLC level in nasal secretions higher than 34.243 ng/mL can predict postoperative polyp recurrence of CRSwNP, which is associated with refractory CRSwNP (36, 37). ECP and eotaxin, as markers of EOS, have been suggested as predictors of postoperative recurrence in patients with CRSwNP (38), and eotaxin-3 expression may also be an important marker for ECRSwNP typing and the prediction of disease severity in ECRSwNP patients (**Figure 1**) (**Table 1**).

### 3.2 Non-type 2 inflammatory factors

Gao et al. (18) reported that in patients with ECRSwNP, higher IL-6 levels (AUC = 0.707) could increase the risk of postoperative recurrence and have good value for predicting postoperative recurrence (**Table 1**). Findings from some studies have suggested that transforming growth factor-β1 (TGF-β1), matrix metalloproteinase 9 (MMP-9), tissue inhibitor of metalloproteinase 1 (TIMP-1), IL-1, IL-4, IL-6, IL-17 and tumor necrosis factor-α (TNF-α) are factors influencing the development of refractory CRS (39). IL-36α may be a biomarker for predicting refractory CRSwNP in Asians (40). Xie et al. (41) reported that chemerin and IL-17 are potential predictors of CRS and that chemerin silencing can reduce inflammation and bone remodeling in CRS patients. Hussien et al. (42) reported that an IL-17 concentration greater than 9.2 pg/ml and a pentraxin-3 concentration greater than 4.12 ng/ml were considered important biological indicators with high sensitivity and specificity for the diagnosis and follow-up of CRSwNP patients (**Table 1**). Tang et al. (43) reported that IL-22 and IL-17A may play important roles in regulating T helper type 2 (Th2) inflammatory skewing in AR patients (**Figure 1**).

## 4 Inflammatory cell markers

### 4.1 EOSs

EOSs are key effector cells that affect the prognosis of type 2 CRS, and EOS infiltration is a characteristic feature observed in most CRSwNP patients. At least 1 year after surgery, a tissue EOS count greater than 48/HPF, a blood EOS percentage greater than 2.5% or a tissue EOS percentage greater than 20.6% were independent risk factors for uncontrolled CRS (6) (**Table 1**). Lou et al. (44) reported that a tissue EOS count greater than 55/HPF or an EOS ratio greater than 27% could predict the recurrence of nasal polyps (**Table 1**). Brescia et al. (45) reported that increased serum EOS and basophil counts were associated with an increased risk of recurrence of nasal polyps, and the likelihood of developing nasal polyps in patients with a serum EOS percentage ≥ 3.7% was more than twice that of patients with a serum EOS percentage < 3.7% (**Table 1**). Other studies have demonstrated that the presence of EOSs in tissue or blood is significantly correlated with the development or recurrence of CRSwNP (20) and may be significantly associated with olfactory dysfunction (46). A study on the role of blood EOS and basophil counts in predicting AFRS revealed that the EOS and basophil counts in patients with recurrent AFRS were significantly greater than those in patients without recurrence of AFRS (47). However, there is currently no consensus on the tissue or serum EOS count thresholds and their effects on CRS patient outcomes. Large-scale multicenter studies should be performed to establish a widely acceptable EOS cutoff to predict definite treatment outcomes (**Figure 1**).

### 4.2 Neutrophils

The importance of neutrophils in the pathogenesis of CRS has received increasing attention. Some studies have shown that a high level of subepithelial neutrophil infiltration may be used as a cellular biomarker of refractory CRSwNP (48). Kim et al. (40) revealed that in South Korean patients with CRSwNP, at 1 year after surgery, a subepithelial human neutrophil elastase (HNE)-positive neutrophil number greater than 45/HPF could be used as a cell marker of refractory CRS (**Table 1**). Cha et al. (49) reported that tissue neutrophilia and neutrophil extracellular trap formation (NETosis) can serve as prognostic biomarkers for neutrophilic CRSwNP. Subash et al. (50) conducted a prospective study and reported that the neutrophil–lymphocyte ratio (NLR) could be used as a marker for the severity and prognosis of nasal polyps and AFRS. In type 3 CRS, higher levels of neutrophil markers are correlated with disease severity and the CRSwNP phenotype (51) (**Figure 1**).

## 5 Metabolites

### 5.1 Fatty acid (arachidonic acid and linoleic acid) metabolism

**Figure 2:**
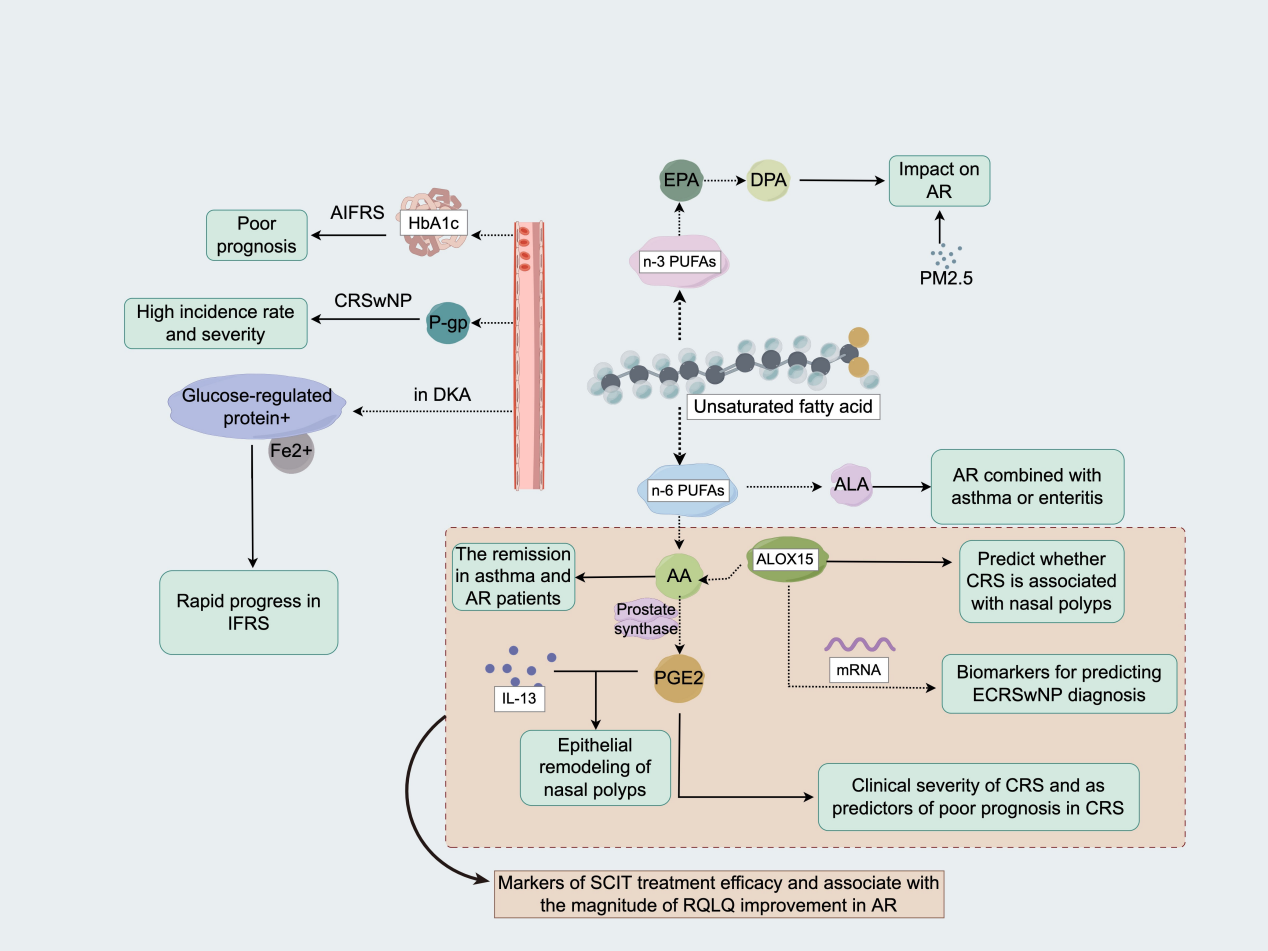
Role of metabolic product predictive indicators in the adverse outcomes of nasal inflammatory diseases (1) Fatty acid metabolism: The DPA of EPA in n-3 PUFAs can counteract the impact of PM2.5 on AR. AA and its downstream metabolites in n-6 PUFAs can serve as therapeutic efficacy biomarkers for the SCIT of AR and are related to the degree of improvement in the RQLQ of AR patients. AA is associated with the remission probability of patients with AR and asthma. ALOX15 can be a reliable biomarker for predicting whether CRS is accompanied by nasal polyps and for diagnosing ECRSwNP. PGE2 is a key component of IL-13-induced epithelial remodeling in nasal polyps, is associated with the clinical severity of CRS and can be used as a predictor of poor prognosis in CRS patients. ALA may be a metabolic marker for AR patients with coexisting asthma or enteritis (2). Carbohydrate metabolism: P-gp is associated with a high incidence and can be used as an indicator of disease severity in patients with CRSwNP. Serum iron and overexpressed glucose-regulated protein in patients with diabetic ketoacidosis can lead to rapid progression of IFRS. Glycated HbA1c can be used as a marker for a poor prognosis of AIFRS. DPA, product docosapentaenoic acid; EPA, eicosapentaenoic acid; PUFAs, polyunsaturated fatty acids; PM 2.5, particulate matter **≤**2.5 µm in size; AR, allergic rhinitis; AA, arachidonic acid; SCIT, subcutaneous immunotherapy; RQLQ, rhinoconjunctivitis quality of life questionnaire; ALOX15, arachidonate 15-lipoxygenase; CRS, chronic rhinosinusitis; ECRSwNP, eosinophilic chronic rhinosinusitis with nasal polyps; PGE2, prostaglandin E2; IL, interleukin; ALA, α-linoleic acid; P-gp, p-glycoprotein; CRSwNP, chronic rhinosinusitis with nasal polyps; IFRS, invasive fungal rhinosinusitis; HbA1c, hemoglobin A1c; AIFRS, acute invasive fungal rhinosinusitis.

Polyunsaturated fatty acids (PUFAs) can be used as monitoring markers or novel treatments for AR. PUFAs include n-3 PUFAs (mainly eicosapentaenoic acid (EPA), docosahexaenoic acid (DHA), and linolenic acid (LNA)) and n-6 PUFAs (mainly AA and LA). Docosapentaenoic acid (DPA) is a product of EPA. If pregnant women have high DPA levels in red blood cells, both the women and their offspring are more likely to be protected against the AR response caused by particulate matter ≤2.5 µm in size (PM2.5) (52). Zheng et al. (53) conducted a prospective study and reported that the levels of AA and its downstream metabolites could be used as markers of the efficacy of subcutaneous immunotherapy (SCIT) for AR and that the levels of these metabolites were correlated with improvements in the Rhinoconjunctivitis Quality of Life Questionnaire (RQLQ) score. Magnusson et al. (54) reported that AA levels and the presence of AR were related to the likelihood of achieving remission in asthma patients. Arachidonate 15-lipoxygenase (ALOX15) is a key metabolic enzyme that converts AA into biologically active metabolites, and some studies have shown that ALOX15 expression is upregulated in CRSwNP, where ALOX15 is involved in the recruitment and activation of EOSs; therefore, ALOX15 can be used as a predictive biomarker of CRS in combination with nasal polyps. Moreover, the ALOX15 mRNA level alone (AUC = 0.909) or in combination with the blood EOS count (AUC = 0.933) could be a reliable biomarker for predicting the diagnosis of ECRSwNP (55) (**Table 1**). The prostaglandin (PG) E2 produced in the metabolism of AA is a key component of IL-13-induced epithelial remodeling in nasal polyps, is correlated with the clinical severity of CRS, and can be used as a predictor of poor CRS prognosis (56). Findings from some studies have suggested that α-linoleic acid (ALA) may be a metabolic marker that is predictive of AR complicated with asthma or enteritis (57) (**Figure 2**).

### 5.2 Glucose metabolism

Glucose metabolism has been shown to promote glucose uptake and mainly glycolysis by epithelial cells, which strengthens the proinflammatory function of epithelial cells in CRS (57). A P-glycoprotein (P-gp) secretion level greater than 250 pcg/μg is associated with a high incidence of CRSwNP and can be used as an indicator of disease severity (58) (**Table 1**). Serum iron levels and the overexpression of glucose-regulated proteins can increase the sensitivity of endothelial cells to fungi and induce fungal invasion and injury in patients with diabetic ketoacidosis (59); moreover, the hyperglycemic and acidic status of these cells can lead to the rapid progression of invasive fungal rhinosinusitis (IFRS) (60). Barbara et al. (61) studied the prognostic factors of acute invasive fungal rhinosinusitis (AIFRS) associated with coronavirus disease 2019 (COVID-19) and reported that the level of glycated hemoglobin (HbA1c) can be used as a marker of a poor prognosis (**Figure 2**) (**Table 1**).

## 6 Microorganisms

In nasal inflammatory diseases, nasal homeostasis is usually affected, as an imbalance in nasal commensal microbes is often observed. A reduction in the abundance of protective microbiota or an increase in the abundance of harmful microbiota may disturb the stability of the nasal microbiota, thus facilitating the development of CRSwNP (62); moreover, the abundances of Corynebacterium, Anaerococcus and Thermomonas were associated with surgical outcomes and could predict recurrence, and the predictive performance of the abundances of these genera was even better than that of prediction models based on clinical features (62). Compared with those of healthy patients, the nasal microbiomes of ECRSwNP patients exhibit greater species richness and homogeneity, and these nasal microbiota metrics are correlated with the severity of ECRSwNP (63). Some studies have shown that during skull base surgery or endoscopic sinus surgery, a mixture of fungi and bacteria located in sinus tissues may be an indicator of a poor prognosis of CRS, and CRS may develop into IFRS under certain conditions (13). In AR, inflammatory response features may affect the nasal microbiota, and the nasal microbiota has the potential to be used as a candidate biomarker for the diagnosis of AR (64). In summary, changes in the characteristics of the nasal microbiota may be predictive of poor prognosis for nasal inflammatory diseases (**Figure 1**).

## 7 Others

### 7.1 Clinical imaging

Studies have revealed that the threshold ratio of total ethmoid sinus (E) and total maxillary (M) scores (E/M ratio) for both sides of the Lund–Mackay computed tomography (CT) score has the highest predictive value for the diagnosis of eosinophilic chronic rhinosinusitis (ECRS) and the recurrence of CRSwNP (65); when the threshold ratio of total ethmoid sinus (E) and total maxillary (M) scores (E/M) is greater than 2.55, the likelihood of CRSwNP recurrence is increased (66) (**Table 1**). In another study on the development of a radiomic identification model for ECRSwNP, a CT radiomic model based on 10 radiomic features was found to be promising for the identification of ECRSwNP, and this model may provide new insights to address other clinical issues, such as guiding personalized treatment and predicting CRSwNP patient outcomes (67). In a magnetic resonance imaging (MRI) study on vision and mortality outcomes associated with AIFRS, when orbital apex or cerebral artery involvement or both were present, the findings of MRI performed before the initial intervention could predict poor visual outcomes; facial soft tissue, the nasolacrimal duct, intracranial involvement or a combination of these three were associated with an increased risk of death, and hard palate involvement was associated with a poor prognosis (68). A study in AIFRS revealed that if the extranasal lesion with loss of contrast enhancement (LoCE) found on Gd-enhanced MR images could not be completely removed by surgery, the prognosis might be poor (69). Among the various other clinical and radiological factors, the LoCE was an independent prognostic factor (70). Clinical imaging is a noninvasive and efficient means to predict the development of nasal inflammatory diseases, but the accuracy of imaging-based predictions still needs to be confirmed through additional studies.

### 7.2 Other clinically relevant factors

Studies have revealed that CRSwNP patients with asthma, high Lund–Kennedy scores, high visual analog scale (VAS) scores and high olfactory loss scores are more likely to recur (66). In a clinical study of indicators of IFRS outcomes in children, survival was determined by the absolute neutrophil count, recovery time, brain involvement, fungal types, patient condition in the intensive care unit (ICU), and hyperglycemia (71). A study on the survival of patients with AIFRS revealed that the shorter the duration of prediagnostic symptoms was, the worse the overall survival of patients with AIFRS (72). Owing to the variety of clinical symptoms of nasal inflammatory diseases and large individual differences, the use of a clinical score to predict the poor prognosis of nasal inflammatory diseases is controversial. In the future, a joint prediction model of clinical scores together with other predictors may be established to prevent unfavorable outcomes of nasal inflammatory diseases.

### 7.3 Application of AI

Some studies have shown that a naive Bayes model based on the combination of environmental PM exposure and the EOS count can predict the risk of CRSwSP recurrence (73). In reference to existing international diabetes management programs, Bousquet et al. (74) proposed a model in which the MASK-air app was used to manage AR patient symptoms according to an electronic daily Combined Symptom and Medication Score (CSMS) or an electronic Daily Control Score for Asthma (e-DASTHMA). The AI Chronic Rhinosinusitis Evaluation Platform 2.0 (AICEP 2.0) was established to determine the proportion of inflammatory cells in the clinical diagnosis of nasal polyp cells and to explore the clinical significance of different nasal polyp phenotypes (75). In addition, a multitask deep learning network based on a deep learning radiology nomogram can be used for sinus segmentation and noninvasive prediction of CRS recurrence (65).

Although the aforementioned AI applications for predicting poor outcomes of nasal inflammatory diseases are promising, their inherent limitations and risks in clinical translation warrant serious attention, with drawbacks being highly intertwined with the core research challenges in this field. At the data foundation level, several core AI models (65, 73) are trained on single-center homogeneous cohorts or are restricted to specific detection equipment, resulting in limited generalizability of the models. In terms of functional limitations, the phenotypic diagnosis platform (75) lacks longitudinal validation capabilities, as it does not meet the research needs for assessing dynamic changes in biomarkers; the symptom management tool (74) relies on patient-reported data with quality biases, which may trigger cascading risks if directly used as input for prognostic models.

## 8 Advances in the treatment of nasal inflammatory diseases through drugs targeting poor prognostic indicators

### 8.1 Targeted monoclonal antibody therapy

Although medical and surgical treatments for nasal inflammatory diseases are currently available, the treatment effects remain unsatisfactory; therefore, an increasing number of studies have been conducted on targeted biological therapy for nasal inflammatory diseases. Targeting type 2 inflammatory cytokines, such as IL-4, IL-5, IL-13, and IgE, is currently considered a promising treatment approach. Through indirect comparisons of placebo-controlled trials, mepolizumab (anti-IL-5), reslizumab (anti-IL-5), benralizumab (anti-IL-5Rα), omalizumab (anti-IgE), and dupilumab (anti-IL-4Rα, inhibition of IL-4 and IL-13 signal transduction) have been shown to reduce nasal airway obstruction in patients with CRSwNP and to reduce the likelihood of requiring emergency medical treatment and/or surgical removal of polyps (76–79) (**Table 2**).

**Table 2:** Targeted monoclonal antibody drugs for nasal inflammatory diseases.

Drug	Target	Results	Advantages	Limitations	References
Mepolizumab	IL-5	Reduced the surgical needs of patients with severe recurrent bilateral nasal polyps and systemic type 2 inflammatory markers, and reduced the nasal polyp and nasal congestion scores of patients by targeting IL-5	·It can alleviate symptoms of nasal congestion and loss of smell ·FDA has approved it for the treatment of patients aged 18 years and older with CRSwNP	Mild adverse events such as allergic reaction, bronchitis, dyspnea, headache, infection, mild increase in thyroid hormones, nasopharyngitis, and ear pain, etc., may occur	(77, 78, 80, 82, 83, 85)
Reslizumab	IL-5	By specifically targeting and binding to IL-5, reduced the EOS count, nasal and peripheral IL-5, soluble IL-5Rα and ECP levels in patients with CRSwNP	Target IL-5 with afilinity and specificity	·Upper respiratory tract infection is prone to occur ·Clinical trials are ongoing and it is not yet approved for clinical use	(76, 82)
Benralizumab	IL-5Rα	·Was shown to act on IL-5α and reduce the nasal polyp, nasal obstruction and olfactory scores of CRSwNP patients ·It was beneficial for the control of asthma	·Leading to EOS death and a reduction in inflammation ·Alleviates symptoms of nasal congestion and loss of smell	May lead to adverse events, such as asthma, fever, headache, nasopharyngitis, otitis media, sinusitis and urinary tract infection, etc.	(77, 82, 86, 87)
Omalizumab	IgE	·Improved the clinical symptoms, SNOT-22 score and nasal polyp score of CRSwNP, and reduced the levels of serum periostin, ECP and soluble IL-5Rα by targeting IgE ·Improved the daily nasal and ocular symptom scores, medication symptom scores, and the proportion of emergency medication days in AR patients, and the safety of immunotherapy for AR	It is an FDA-approved anti-IgE biologic for the treatment of CRSwNP and the most effective agent in improving the ear/facial pain subdomain of SNOT-22	May lead to allergic reaction, asthma, common cold, headache, left ulnar hypoesthesia, nasal obstruction, otitis media, and shortness of breath, etc.	(77, 78, 80, 82, 88–92)
Dupilumab	IL-4Rα	·By blocking IL-4 and IL-13 signal transduction, improved the outcomes, symptoms and nasal polyp scores of patients with severe CRSwNP and refractory ECRS, and reduced the levels of type 2 biomarkers ·Significantly alleviated AR-related symptoms in patients with comorbid asthma	·Has been approved for the treatment of CRSwNP ·As an essential component for the treatment of patients with severe CRSwNP and refractory ECRS ·May be a valuable option for patients with AR uncontrolled by conventional therapies	May lead to back pain, dizziness, epistaxis, headache, injection site reaction, nasopharyngitis, oropharyngeal pain, and upper respiratory tract infection, etc.	(77–79, 82, 93–100)
CM310	IL-4Rα	·By targeting and binding to IL-4α, it thereby reduced the size of nasal polyps, improved the symptom scores and enhanced the quality of life of patients with severe ECRSwNP	·The incidence of adverse events was low ·The alternative treatment options for patients with an inadequate response to or contraindications for dupilumab	The reported adverse events include tinnitus, upper respiratory tract infection, and increased serum cholesterol, etc.	(82, 102)
Tezepelumab	TSLP	·By targeting TSLP, it acted to improve the efficacy of subcutaneous immunotherapy for AR patients ·Alleviated symptoms in patients with severe, uncontrolled asthma and a history of CRSwNP	·The alternative treatment options for patients with an inadequate response to or contraindications for dupilumab ·Delays surgery time of nasal polyp	Adverse events include back pain, epistaxis, headache, nasopharyngitis, and upper respiratory tract infection, etc.	(77, 82, 101)

IL, interleukin; FDA, the U.S. Food and Drug Administration; CRSwNP, chronic rhinosinusitis with nasal polyps; EOS, eosinophil; IgE, immunoglobulin E; SNOT-22, 22-item sino-nasal outcome test; ECP, eosinophil cationic protein; ECRS, eosinophilic chronic rhinosinusitis; AR, allergic rhinitis; ECRSwNP, eosinophilic chronic rhinosinusitis with nasal polyps; TSLP, thymic stromal lymphopoietin.

Numerous studies have revealed that mepolizumab and reslizumab, which bind to IL-5 with high affinity and specificity, can reduce the EOS count, nasal and peripheral IL-5 levels, soluble IL-5Rα levels, and ECP levels in CRSwNP patients (80–82). The anti-IL-5 monoclonal antibody mepolizumab has been shown to reduce the need for surgical intervention in patients with severe, recurrent bilateral nasal polyps; improve nasal polyp size and nasal congestion severity scores; and decrease systemic levels of type 2 inflammatory markers, including IgE, periostin, MMP-9, myeloperoxidase, PGD2, PGF2α, LTB4, and thrombin (77, 80, 83–85). A phase III randomized study of benralizumab (anti-IL-5Rα antibody) revealed that, compared with placebo, benralizumab could reduce the nasal polyp score, nasal obstruction score and olfactory score in patients with CRSwNP; furthermore, benralizumab was beneficial for the control of asthma symptoms (77, 82, 86, 87). Accordingly, serum IL-5 levels serve as a key predictive biomarker for anti-IL-5/IL-5Rα therapy with mepolizumab, reslizumab, and benralizumab. Numerous studies have shown that omalizumab can alleviate clinical symptoms; reduce the SNOT-22 score and nasal polyp score; and reduce serum periostin, ECP and soluble IL-5Rα levels in CRSwNP patients (77, 78, 80, 88, 89). In AR patients, omalizumab reduced the daily nasal and eye symptom scores, the medication symptom score, and the proportion of days of emergency drug use and improve the safety of immunotherapy for AR (90, 91). The application of this therapy is specifically guided by elevated serum IgE levels, establishing IgE as its primary predictive biomarker (82, 92). Multiple studies have shown that a dupilumab +/-mometasone nasal spray can improve outcomes, alleviate symptoms and reduce nasal polyp scores in patients with severe CRSwNP and refractory ECRS (77–79, 93–95); furthermore, this combined treatment can decrease the levels of type 2 biomarkers (such as eotaxin-2, ECP, IL-5, IL-13, and IgE) and significantly alleviate AR-related symptoms in patients with comorbid asthma (82, 96–100). Therefore, the efficacy of dupilumab is correlated with a broader type 2 inflammatory signature, and biomarkers such as IL-4, IL-1, eosinophils, and IgE can inform its predictive value. Tezepelumab can increase the efficacy of SCIT in patients with AR (101). Shen et al. (102) reported that CM310, a monoclonal antibody targeting IL-4Rα, was safe and effective at reducing the size of nasal polyps, improving symptom scores, and improving the QoL of patients with severe ECRSwNP. The IL-4Rα chain (also known as CD124) is the major component of the IL-4 receptor (103). Injections of an anti-CD124 monoclonal antibody (αCD124) have long been used to treat CRSwNP (101). Wu et al. (104) reported that the use of protamine nanostructures to encapsulate αCD124 led to increased efficacy of the localized intranasal administration of αCD124, which revealed that the local administration of drugs via nanomaterials may become a future research direction.

### 8.2 Treatments to improve the nasal microbial environment (antibiotics, probiotics, and antifungal drugs)

In view of the influence of the microbiota on nasal inflammatory diseases, changing the characteristics of the nasal microbial environment may prevent negative clinical outcomes of nasal inflammatory diseases. According to the 2020 European document on rhinosinusitis and nasal polyps, antibiotics (usually macrolides) are often used to treat CRS to change the characteristics of the nasal microbial environment (105). Some studies have shown that probiotics can relieve the symptoms of CRS and prevent poor prognoses (106), and the topical use of probiotics prevented infection by Corynebacterium tuberculostearicum in a CRS mouse model (107). Since probiotics are less harmful and effective at alleviating AR symptoms and improving QoL (107), in the International Consensus Statement on Allergy and Rhinology: Allergic Rhinitis-2023, it is recommended that probiotics be used as adjuvant therapy for symptomatic seasonal or perennial AR (108). For fungal infections in patients with IFRS, the current antifungal treatments are oral amphotericin B lipid preparations and voriconazole (109). However, the use of antibiotics is controversial because of the wide range of microbial populations involved in nasal inflammatory diseases. Owing to the individualized efficacy of probiotic treatment, large-scale studies are needed to confirm the efficacy of changing the nasal microbial environment as a treatment.

### 8.3 Others (hormones, antihistamines, immunotherapy, lipid metabolites, and traditional Chinese medicines)

Other treatments for nasal inflammatory diseases include hormones, antihistamines, and immunotherapy, and hormones and antihistamines are often used to control symptoms. Hormones are beneficial for the control of olfactory dysfunction in CRS patients (110). Some scholars believe that steroid hormones can control the allergic response of AR patients by inhibiting Th2 cytokines (IL-4, IL-5, IL-13, etc.) (111). H1 antihistamines (such as chlorpheniramine, loratadine, and fexofenadine) and intranasal antihistamines (such as azelastine and olopatadine) can reduce the expression of proinflammatory cytokines and cell adhesion molecules; therefore, they are often used to treat AR (112). Treatment with loratadine combined with glucocorticoids has been confirmed to reduce the incidence of adverse reactions and nasal symptom scores in children with AR (113). Allergen immunotherapy (AIT) is a treatment for AR and has a long history of clinical application. The AITs used for AR mainly include SCIT, sublingual immunotherapy (SLIT), intralymphatic immunotherapy (ILIT), and local immunotherapy. SCIT can reduce the symptom score, drug score, disease-specific QoL score and serum sIgG4 level of AR patients (114). SLIT is important for preventing asthma development or reducing the risk of asthma exacerbation in AR patients (115). The use of ILIT helps relieve rhinitis symptoms, reduce drug dosage, and reduce the VAS score and RQLQ score (116).

As a metabolite of EPA, 15-hydroxyeicosapentaenoic acid (15-HEPE) has also been shown to be an EOS-dependent antiallergic metabolite that can be applied in the prevention and treatment of AR (117). ALA was shown to significantly relieve nasal symptoms, reduce serum OVA-sIgE levels, and correct the Th1/Th2 imbalance in AR mice (118). Short-chain fatty acid (SCFA) supplementation or SCFA-producing bacteria administration helps relieve AR symptoms (119). Alfalfa (*Medicago sativa*) was used in AR treatment because of its anti-inflammatory effects and ability to inhibit key enzymes involved in AA metabolism.

TCMs such as crocin can reduce the levels of Th2 cytokines such as IL-4 and inhibit the NF-κB signaling pathway, thereby inhibiting inflammation in ECRS (120). Xiao-qing-long-tang (a TCM) was shown to relieve nasal symptoms and reduce the levels of type 2 cytokines and OVA-sIgE in AR mice, thereby reducing type 2 inflammation in AR (121).

The role of dietary factors in managing chronic diseases like type 2 diabetes has been widely explored. Just as specific interventions are used for glycemic control in diabetic patients, predictive biomarkers in inflammatory diseases can help stratify patient risk and optimize therapeutic approaches. Biomarkers, much like dietary interventions, serve as a tool for personalized treatment, improving patient outcomes through targeted approaches (122).

## 9 Challenges and future perspectives

Biomarkers for nasal inflammatory diseases have well-documented clinical value, but their application is limited due to significant standardization barriers. Core limitations include the lack of standardized cutoff values, the heterogeneity of the patient population, and overlooked endotypic stratification (10, 18, 51). Additional research gaps include insufficient longitudinal data on the dynamic changes in biomarkers during treatment and inadequate research in special populations (28, 30). Although some clinical applications of targeted mAbs against specific biomarkers and some biomarkers, such as IgE, IL-5, EOSs, and the E/M ratio, are expected to predict postoperative recurrence risk, the intrinsic type, and the therapeutic effect of CRSwNP (**Table 3**), many clinical trials are still needed to verify their value (6, 16, 22, 44, 66).

**Table 3:** The most promising predictive markers.

Type	Marker	Associated disease	Predictive value for specific outcome	Key references
Type 2 inflammatory factors	sIgE	CRSwNP	Predicts the recurrence of CRSwNP after surgery	(18)
	IL-5	CRSwNP	Predicts the postoperative recurrence of CRSwNP	(22)
	IL-33	AR and CRSwNP	·Early diagnosis of AR ·Predicts the postoperative recurrence of CRSwNP	(23, 24)
	TSLP	AR and CRSwNP	Predicts the diagnosis of AR and postoperative outcomes in CRSwNP patients	(25, 26)
	Periostin	CRS	Predicts the postoperative recurrence of CRS, especially CRSwNP	(28)
	CST1	CRSwNP	Predicts the recurrence of CRSwNP after surgery	(34)
	CLC	CRSwNP	Predicts the postoperative recurrence of CRSwNP and is associated with refractory CRSwNP	(36)
Inflammatory cell markers	EOSs	CRSwNP	Predicts the recurrence of CRSwNP and olfactory dysfunction	(45)
	HNE-positive neutrophils	CRSwNP	Predicts the refractory CRSwNP	(40)
	NLR	AFRS	Marker for the severity and prognosis of AFRS	(50)
Metabolism-related biomarker	ALOX15	ECRSwNP	Predicts the diagnosis of ECRSwNP	(55)
Clinical imaging	E/M ratio	CRSwNP	Predicts the recurrence of CRSwNP	(65)

sIgE, specific immunoglobulin E; CRSwNP, chronic rhinosinusitis with nasal polyps; IL, interleukin; ECRSwNP, eosinophilic chronic rhinosinusitis with nasal polyps; AR, allergic rhinitis; CRS, chronic rhinosinusitis; TSLP, thymic stromal lymphopoietin; CST1, cystatin SN; CLC, Charcot-Leyden Crystal; EOS, eosinophil; NLR, neutrophil-lymphocyte ratio; AFRS, acute fungal rhinosinusitis; ALOX15, arachidonate 15-lipoxygenase; E/M ratio, the threshold ratio of total ethmoid sinus (E) and total maxillary (M) scores.

Currently, a multiparameter integration framework (incorporating molecular biomarkers, clinical indicators, and imaging features) has emerged as a potential solution to the aforementioned issues. However, this framework still has unresolved limitations and research gaps: First, endotypic stratification in biomarker development is overly simplistic—existing studies rarely distinguish between eosinophilic, neutrophilic, or mixed subtypes of CRS, resulting in “one-size-fits-all” biomarkers with poor specificity for subtype-specific poor prognoses (10, 51, 63); second, the “translation gap” between preclinical biomarker discovery and clinical application persists, as standardized detection protocols and validation in real-world clinical settings are lacking for many promising candidates (12, 34); third, previous studies have failed to integrate dynamic multidimensional data, such as longitudinal changes in biomarkers during treatment and patient-reported outcomes, leading to incomplete prognostic assessments (28, 74). Notably, AI research in nasal inflammatory diseases also faces bottlenecks: most models rely on single-modal data (e.g., imaging data alone or laboratory indices alone) rather than fusing multisource data, and few models combine phenotypic features to improve prediction accuracy, which limits their clinical utility (65, 75).

Future research should focus on three core directions: First, subtype-specific biomarker cutoffs should be established on the basis of multicenter cohorts, exclusive biomarker panels for different CRS subtypes should be developed, and diagnostic and prognostic thresholds should be optimized by integrating individual characteristics such as patient age and comorbidities, thereby enhancing the clinical applicability of biomarkers from the source (18, 23, 67). Second, the dynamic change patterns of biomarkers during treatment should be clarified through longitudinal studies, and key monitoring nodes in different treatment phases should be identified to provide real-time evidence for clinical treatment adjustment (28, 30). Third, the in-depth integration of AI technology and clinical platforms should be vigorously promoted, which represents the most promising direction for future development: develop large AI models based on multimodal data (biomarkers + endoscopic imaging + clinical medical records), embed validated AI models into electronic medical record systems to realize real-time assessment and early warning of recurrence risk, and ensure the stability of models in diverse scenarios, such as primary hospitals, through cross-institutional and cross-regional multicenter validation (65, 67, 75).

## 10 Conclusion

Inflammatory cytokines, inflammatory cells, metabolites, nasal microbiota, and clinical parameters play significant roles in predicting the prognosis of nasal inflammatory diseases. The combination of AI with clinical indicators and parameters of nasal inflammatory diseases has also been studied to some extent, but further exploration is needed to apply such digital models to the clinical management of the prognosis of nasal inflammatory diseases. Targeted monoclonal antibody drugs such as mepolizumab, reslizumab, benralizumab, omalizumab, dupilumab, and tezepelumab, which target type 2 inflammatory factors, have been shown to significantly improve systemic type 2 biomarkers, nasal symptoms, nasal polyps, and clinical symptom scores in patients, making them promising treatment options for nasal inflammation. Probiotics are currently considered for the adjunctive treatment of AR, but their efficacy varies greatly among individuals. Additionally, the use of antibiotic drugs in nasal inflammatory diseases remains controversial. Therefore, further exploration is needed on how to treat nasal inflammatory diseases by improving nasal microecology. Corticosteroids and antihistamines are drugs commonly used to control allergic reactions in patients with AR, and their combined use has been proven to reduce the incidence of adverse reactions and nasal symptom scores. AIT has been shown to alleviate rhinitis symptoms and improve clinical scores. Metabolic products such as 15-HEPE, ALA, and SCFAs are believed to be applicable for the prevention and treatment of AR. Studies have shown that traditional Chinese medicines such as crocin and Xiao-qing-long-tang can improve the inflammatory response in ECRS and AR and may become new drugs for the treatment of nasal inflammatory diseases in the future.

## Glossary

15-HEPE: 15-hydroxyeicosapentaenoic acid
αCD124: anti-CD124 monoclonal antibody
AA: arachidonic acid
AFRS: allergic fungal rhinosinusitis
AIFRS: acute invasive fungal rhinosinusitis
AICEP 2.0: AI Chronic Rhinosinusitis Evaluation Platform 2.0
AI: artificial intelligence
AIT: Allergen immunotherapy
ALA: α-linoleic acid
ALOX15: arachidonate 15-lipoxygenase
anti-dsDNA: anti-double-stranded DNA
AR: allergic rhinitis
AUC: area under the curve
CLC: Charcot-Leyden Crystal
CSMS: combined Symptom and Medication Score
CST1: cystatin SN
COVID-19: coronavirus disease 2019
CRS: chronic rhinosinusitis
CRSwNP: chronic rhinosinusitis with nasal polyps
CT: computed tomography
DHA: docosahexaenoic acid
DPA: docosapentaenoic acid
ECP: eeosinophil cationic protein
ECRS: eosinophilic chronic rhinosinusitis
ECRSwNP: eosinophilic chronic rhinosinusitis with nasal polyps
E/M ratio: the threshold ratio of total ethmoid sinus (E) and total maxillary (M) scores
EOS: eosinophil
EPA: eicosapentaenoic acid
FDA: the U.S. Food and Drug Administration
FRS: fungal rhinosinusitis
HbA1c: hemoglobin A1c
IFRS: invasive fungal rhinosinusitis
HNE: human neutrophil elastase
HPF: high-power fields
ICU: intensive care unit
IL: interleukin
ILIT: intralymphatic immunotherapy
LA: linoleic acid
LNA: linolenic acid
LoCE: loss of contrast enhancement
MeSH: Medical Subject Headings
MMP-9: matrix metalloproteinase 9
MRI: magnetic resonance imaging
NETosis: neutrophil extracellular trap formation
NLR: neutrophil-lymphocyte ratio
PG: prostaglandin
P-gp: P-glycoprotein
PM2.5: particulate matter ≤2.5 µm in size
PRISMA: Preferred Reporting Items for Systematic Reviews and Meta-Analyses
PUFAs: polyunsaturated fatty acids
QoL: quality of life
RQLQ: Rhinoconjunctivitis Quality of Life Questionnaire
SCFA: short-chain fatty acid
SCIT: subcutaneous immunotherapy
sIgE: specific immunoglobulin E
SLIT: sublingual immunotherapy
SNOT-22: 22-item sino-nasal outcome test
sST2: soluble ST2
TGF-β1: transforming growth factor-β1
Th2: T helper type 2
TCMs: traditional Chinese medicines
TIMP-1: tissue inhibitor of metalloproteinase 1
TNF-α: tumor necrosis factor-α
TSLP: thymic stromal lymphopoietin
VAS: visual analog scale.

## References

[1] Shi JB Fu QL Zhang H Cheng L Wang YJ Zhu DD . Epidemiology of chronic rhinosinusitis: Results from a crosstsnusitis: survey in seven chinese cities. Allergy. (2015) 70:533–9. doi: 10.1111/all.12577, PMID: 25631304 PMC4409092

[2] Meltzer EO . Allergic rhinitis: Burden of illness, quality of life, comorbidities, and control. Immunol Allergy Clin North Am. (2016) 36:235–48. doi: 10.1016/j.iac.2015.12.002, PMID: 27083099

[3] Wang XD Zheng M Lou HF Wang CS Zhang Y Bo MY . An increased prevalence of selfalence6/j allergic rhinitis in major chinese cities from 2005 to 2011. Allergy. (2016) 71:1170–80. doi: 10.1111/all.12874, PMID: 26948849 PMC5074323

[4] Zhang J Li Y Lu X Wang X Zang H Wang T . Analysis of fungal ball rhinosinusitis by culturing fungal clumps under endoscopic surgery. Int J Clin Exp Med. (2015) 8:5925–30., PMID: 26131186 PMC4483880

[5] Thanaviratananich S Cho S-H Ghoshal AG Muttalif ARBA Lin H-C Pothirat C . Burden of respiratory disease in Thailand. Med (Baltim). (2016) 95:e4090. doi: 10.1097/md.0000000000004090, PMID: 27428193 PMC4956787

[6] Tao X Chen F Sun Y Wu S Hong H Shi J . Prediction models for postoperative uncontrolled chronic rhinosinusitis in daily practice. Laryngoscope. (2018) 128:2673–80. doi: 10.1002/lary.27267, PMID: 30295929

[7] Nappi E Paoletti G Malvezzi L Ferri S Racca F Messina MR . Comorbid allergic rhinitis and asthma: Important clinical considerations. Expert Rev Clin Immunol. (2022) 18:747–58. doi: 10.1080/1744666x.2022.2089654, PMID: 35695326

[8] Moghadam MN Amiresmaili M Goudarzi R Amini S Khosravi S . Investigating the appropriateness of admission and hospitalization at a teaching hospital: A case of a developing country. Iran J Public Health. (2017) 46:1720–5., PMID: 29259948 PMC5734973

[9] Hao Y Yang Y Zhao H Chen Y Zuo T Zhang Y . Multi-omics in allergic rhinitis: Mechanism dissection and precision medicine. Clin Rev Allergy Immunol. (2025) 68:19. doi: 10.1007/s12016-025-09028-3, PMID: 39964644 PMC11836232

[10] Wang M Li Y Li J Yan B Wang C Zhang L . New insights into the endotypes of chronic rhinosinusitis in the biologic era. J Allergy Clin Immunol. (2025) 156:51–60. doi: 10.1016/j.jaci.2025.02.015, PMID: 39986619

[11] de Mezer M Chalama N Bratt C Kiebalo M Dolata N Rogalinski J . Changes in the microbiome during chronic rhinosinusitis. Pathogens. (2024) 14:14. doi: 10.3390/pathogens14010014, PMID: 39860975 PMC11768233

[12] Mishra T Sasanka KK Sudha Ty S Kumar D Ps S Bano G . Emerging novel biomarkers in allergic rhinitis: A narrative review. Cureus. (2025) 17:e84705. doi: 10.7759/cureus.84705, PMID: 40551926 PMC12183333

[13] Shin SH Ye MK Lee DW Geum SY . Immunopathologic role of fungi in chronic rhinosinusitis. Int J Mol Sci. (2023) 24:2366. doi: 10.3390/ijms24032366, PMID: 36768687 PMC9917138

[14] Liu S Li J Zhang Y Wang C Zhang L . Il-10: The master immunomodulatory cytokine in allergen immunotherapy. Expert Rev Clin Immunol. (2025) 21:17–28. doi: 10.1080/1744666X.2024.2406894, PMID: 39323099

[15] Yan B Ren Y Liu C Shu L Wang C Zhang L . Cystatin sn in type 2 inflammatory airway diseases. J Allergy Clin Immunol. (2023) 151:1191–203 e3. doi: 10.1016/j.jaci.2023.02.005, PMID: 36958985

[16] Chiu J Fastenberg J Tong C Navetta-Modrov B Marcus S . Biologics for chronic rhinosinusitis with nasal polyposis: Current landscape and future directions. Laryngoscope Investig Otolaryngol. (2025) 10:e70282. doi: 10.1002/lio2.70282, PMID: 41112679 PMC12535206

[17] Mortuaire G Gengler I Carpentier C Szymanski C Chenivesse C Lefevre G . T helper 2 inflammatory markers are associated with recurrence in chronic rhinosinusitis with nasal polyps after endoscopic sinus surgery. Rhinology. (2020) 58:444–50. doi: 10.4193/Rhin19.439, PMID: 32369537

[18] Gao X Zhang J Li A Ding Y Zhao B Wang Y . The value of combined detection of specific immunoglobulin e, interleukin-6 and regulatory t cells in predicting the risk of postoperative recurrence in patients with eosinophilic chronic rhinosinusitis and nasal polyps. J Med Biochem. (2024) 43:537–44. doi: 10.5937/jomb0-48780, PMID: 39139176 PMC11318900

[19] Zielińska-Bliźniewska H Paprocka-Zjawiona M Merecz-Sadowska A Zajdel R Bliźniewska-kowalska K Malinowska K . Serum il-5, postn and il-33 levels in chronic rhinosinusitis with nasal polyposis correlate with clinical severity. BMC Immunol. (2022) 23:33. doi: 10.1186/s12865-022-00507-2, PMID: 35752781 PMC9233770

[20] Rosati D Rosato C Pagliuca G Cerbelli B Della Rocca C Di Cristofano C . Predictive markers of long-term recurrence in chronic rhinosinusitis with nasal polyps. Am J Otolaryngol. (2020) 41:102286. doi: 10.1016/j.amjoto.2019.102286, PMID: 31727332

[21] Zhang Z Liu J Xie L Cao W Ma F Zhang Y . Tissue eosinophils and mucous inflammatory cytokines for the evaluation of olfactory recovery after endoscopic sinus surgery in patients with nasal polyposis. Am J Otolaryngol. (2022) 43:103561. doi: 10.1016/j.amjoto.2022.103561, PMID: 35952528

[22] Bai J Huang JH Price CPE Schauer JM Suh LA Harmon R . Prognostic factors for polyp recurrence in chronic rhinosinusitis with nasal polyps. J Allergy Clin Immunol. (2022) 150:352–61.e7. doi: 10.1016/j.jaci.2022.02.029, PMID: 35305978 PMC9378510

[23] Zhang Y Zhu K Chen J Xia C Yu C Gao T . Predictive values of serum il-33 and sst2 in endotypes and postoperative recurrence of chronic rhinosinusitis with nasal polyps. Mediators Inflamm. (2022) 2022:9155080. doi: 10.1155/2022/9155080, PMID: 35633657 PMC9135518

[24] Jiang Y Hu W Cai Z Lin C Ye S . Peripheral multiple cytokine profiles identified cd39 as a novel biomarker for diagnosis and reflecting disease severity in allergic rhinitis patients. Mediators Inflamm. (2023) 2023:3217261. doi: 10.1155/2023/3217261, PMID: 37207043 PMC10191753

[25] Adhikary PP Tan Z Page BDG Hedtrich S . Tslp as druggable target – a silver-lining for atopic diseases? Pharmacol Ther. (2021) 217:107648. doi: 10.1016/j.pharmthera.2020.107648, PMID: 32758645

[26] Moreno-Jimenez E Morgado N Gomez-Garcia M Sanz C Gil-Melcon M Isidoro-Garcia M . Tslp and tslpr expression levels in peripheral blood as potential biomarkers in patients with chronic rhinosinusitis with nasal polyps. Int J Mol Sci. (2025) 26:1227. doi: 10.3390/ijms26031227, PMID: 39940994 PMC11818291

[27] Xian M Lan F Yan B Shen S Liu S Wan L . An anti-tslp monoclonal antibody for uncontrolled crswnp: The dubhe randomized clinical trial. Nat Commun. (2025) 16:8607. doi: 10.1038/s41467-025-63682-x, PMID: 41022848 PMC12480939

[28] Sato T Ikeda H Murakami K Murakami K Shirane S Ohta N . Periostin is an aggravating factor and predictive biomarker of eosinophilic chronic rhinosinusitis. Allergol Int. (2023) 72:161–8. doi: 10.1016/j.alit.2022.08.006, PMID: 36109310

[29] Kanemitsu Y Kurokawa R Ono J Fukumitsu K Takeda N Fukuda S . Increased serum periostin levels and eosinophils in nasal polyps are associated with the preventive effect of endoscopic sinus surgery for asthma exacerbations in chronic rhinosinusitis patients. Int Arch Allergy Immunol. (2020) 181:862–70. doi: 10.1159/000509253, PMID: 32731246

[30] Ninomiya T Noguchi E Haruna T Hasegawa M Yoshida T Yamashita Y . Periostin as a novel biomarker for postoperative recurrence of chronic rhinosinitis with nasal polyps. Sci Rep. (2018) 8:11450. doi: 10.1038/s41598-018-29612-2, PMID: 30061580 PMC6065353

[31] Yilmaz GO Cetinkaya EA Eyigor H Ellidag HY Balaban K Selcuk OT . The diagnostic importance of periostin as a biomarker in chronic rhinosinusitis with nasal polyp. Eur Arch Otorhinolaryngol. (2022) 279:5707–14. doi: 10.1007/s00405-022-07492-7, PMID: 35723731 PMC9207425

[32] Laury AM Hilgarth R Nusrat A Wise SK . Periostin and receptor activator of nuclear factor κ-c ligand expression in allergic fungal rhinosinusitis. Int Forum Allergy Rhinol. (2014) 4:716–24. doi: 10.1002/alr.21367, PMID: 25060295

[33] Nocera AL Mueller SK Workman AD Wu D McDonnell K Sadow PM . Cystatin sn is a potent upstream initiator of epithelial-derived type 2 inflammation in chronic rhinosinusitis. J Allergy Clin Immunol. (2022) 150:872–81. doi: 10.1016/j.jaci.2022.04.034, PMID: 35660375 PMC9547833

[34] Mueller SK Wendler O Mayr S Traxdorf M Koch M Mantsopoulos K . Comparison of mucus and serum biomarker sampling in chronic rhinosinusitis with nasal polyps. Int Forum Allergy Rhinol. (2023) 14:887–97. doi: 10.1002/alr.23295, PMID: 37990964

[35] Lavin J Min JY Lidder AK Huang JH Kato A Lam K . Superior turbinate eosinophilia correlates with olfactory deficit in chronic rhinosinusitis patients. Laryngoscope. (2017) 127:2210–8. doi: 10.1002/lary.26555, PMID: 28322448 PMC5607065

[36] Chen W Bai Y Kong W Luo X Zeng Y Chen J . Predictive significance of charcotcancela crystal structures for nasal polyp recurrence. Clin Transl Allergy. (2022) 12:e12212. doi: 10.1002/clt2.12212, PMID: 36434740 PMC9679636

[37] Wu D Yan B Wang Y Zhang L Wang C . Predictive significance of charcot-leyden crystal protein in nasal secretions in recurrent chronic rhinosinusitis with nasal polyps. Int Arch Allergy Immunol. (2021) 182:65–75. doi: 10.1159/000510120, PMID: 32927462

[38] Wang G Zheng H Chen X Zheng J Zhan J Li R . Exploration of predictive biomarkers for postoperative recurrence in chronic rhinosinusitis with nasal polyps based on serum multiple-cytokine profiling. Mediators Inflamm. (2022) 2022:1061658. doi: 10.1155/2022/1061658, PMID: 36211987 PMC9534722

[39] Wen W Zhu S Ma R Wang L Shen X Li Y . Correlation analysis of tgf-β1, mmp-9, timp-1, il-1, il-4, il-6, il-17, and tnf-α in refractory chronic rhinosinusitis: A retrospective study. Allergol Immunopathol (Madr). (2022) 50:137–42. doi: 10.15586/aei.v50i4.527, PMID: 35789413

[40] Kim D-K Kim JY Han YE Kim JK Lim H-S Eun KM . Elastase-positive neutrophils are associated with refractoriness of chronic rhinosinusitis with nasal polyps in an asian population. Allergy Asthma Immunol Res. (2020) 12:42–55. doi: 10.4168/aair.2020.12.1.42, PMID: 31743963 PMC6875473

[41] Xie D Bai Z Zhou G Li K Ding J Zhang H . Chemerin and ildme are potential predictors and chemerin silencing alleviates inflammatory response and bone remodeling in chronic rhinosinusitis. Chem Biol Drug Des. (2023) 102:1478–88. doi: 10.1111/cbdd.14339, PMID: 37712455

[42] Hussien HA Habieb MS Hamdan AM . Evaluation of serum total immunoglobulin e, interleukin-17 and pentraxin-3 as biomarkers for chronic rhinosinusitis with nasal polyposis. Am J Rhinol Allergy. (2020) 35:640–6. doi: 10.1177/1945892420983787, PMID: 33356412

[43] Tang J Xiao P Luo X Bai J Xia W Chen W . Increased il-22 level in allergic rhinitis significantly correlates with clinical severity. Am J Rhinol Allergy. (2014) 28:e197–201. doi: 10.2500/ajra.2014.28.4088, PMID: 25514475

[44] Lou H Meng Y Piao Y Wang C Zhang L Bachert C . Predictive significance of tissue eosinophilia for nasal polyp recurrence in the chinese population. Am J Rhinol Allergy. (2015) 29:350–6. doi: 10.2500/ajra.2015.29.4231, PMID: 26219765

[45] Brescia G Barion U Zanotti C Giacomelli L Martini A Marioni G . The prognostic role of serum eosinophil and basophil levels in sinonasal polyposis. Int Forum Allergy Rhinol. (2016) 7:261–7. doi: 10.1002/alr.21885, PMID: 27992119

[46] Hauser LJ Chandra RK Li P Turner JH . Role of tissue eosinophils in chronic rhinosinusitis–associated olfactory loss. Int Forum Allergy Rhinol. (2017) 7:957–62. doi: 10.1002/alr.21994, PMID: 28742240 PMC5624838

[47] Brescia G Franz L Alessandrini L Parrino D Barion U Marioni G . Prognostic role of blood eosinophil and basophil levels in allergic fungal rhinosinusitis (afrs). Am J Otolaryngol. (2020) 41:102301. doi: 10.1016/j.amjoto.2019.102301, PMID: 31732306

[48] Kim DK Lim HS Eun KM Seo Y Kim JK Kim YS . Subepithelial neutrophil infiltration as a predictor of the surgical outcome of chronic rhinosinusitis with nasal polyps. Rhinology. (2021) 59:173–80. doi: 10.4193/Rhin20.373, PMID: 33129200

[49] Cha H Lim HS Park JA Jo A Ryu HT Kim DW . Effects of neutrophil and eosinophil extracellular trap formation on refractoriness in chronic rhinosinusitis with nasal polyps. Allergy Asthma Immunol Res. (2023) 15:94–108. doi: 10.4168/aair.2023.15.1.94, PMID: 36693361 PMC9880302

[50] Subash A Gupta R Gupta A Bansal S Singh A Naseem S . Neutrophil lymphocyte ratio: A predictor of disease severity in nasal polyposis and allergic fungal rhinosinusitis. Indian J Otolaryngol Head Neck Surg. (2022) 74:1128–33. doi: 10.1007/s12070-020-02053-y, PMID: 36452551 PMC9702498

[51] Min J-Y Kim JY Sung CM Kim ST Cho H-J Mun SJ . Inflammatory endotypes of chronic rhinosinusitis in the korean population: Distinct expression of type 3 inflammation. Allergy Asthma Immunol Res. (2023) 15:437–50. doi: 10.4168/aair.2023.15.4.437, PMID: 37075796 PMC10359642

[52] Chen Y Guo C Chung MK Yi Q Wang X Wang Y . The associations of prenatal exposure to fine particulate matter and its chemical components with allergic rhinitis in children and the modification effect of polyunsaturated fatty acids: A birth cohort study. Environ Health Perspect. (2024) 132:47010. doi: 10.1289/ehp13524, PMID: 38630604 PMC11060513

[53] Zheng P Yan G Zhang Y Huang H Luo W Xue M . Metabolomics reveals process of allergic rhinitis patients with single- and double-species mite subcutaneous immunotherapy. Metabolites. (2021) 11:613. doi: 10.3390/metabo11090613, PMID: 34564431 PMC8471092

[54] Magnusson J Ekström S Kull I Håkansson N Nilsson S Wickman M . Polyunsaturated fatty acids in plasma at 8 years and subsequent allergic disease. J Allergy Clin Immunol. (2018) 142:510–6.e6. doi: 10.1016/j.jaci.2017.09.023, PMID: 29221817

[55] Liang Z Yan B Liu C Tan R Wang C Zhang L . Predictive significance of arachidonate 15-lipoxygenase for eosinophilic chronic rhinosinusitis with nasal polyps. Allergy Asthma Clin Immunol. (2020) 16:82. doi: 10.1186/s13223-020-00480-8, PMID: 32973910 PMC7493848

[56] Kotas ME Patel NN Cope EK Gurrola JG Goldberg AN Pletcher SD . Il-13–associated epithelial remodeling correlates with clinical severity in nasal polyposis. J Allergy Clin Immunol. (2023) 151:1277–85. doi: 10.1016/j.jaci.2022.12.826, PMID: 36736797 PMC10243183

[57] Chen Z He S Wei Y Liu Y Xu Q Lin X . Fecal and serum metabolomic signatures and gut microbiota characteristics of allergic rhinitis mice model. Front Cell Infect Microbiol. (2023) 13:1150043. doi: 10.3389/fcimb.2023.1150043, PMID: 37180443 PMC10167002

[58] Nocera AL Meurer AT Miyake MM Sadow PM Han X Bleier BS . Secreted p-glycoprotein is a noninvasive biomarker of chronic rhinosinusitis. Laryngoscope. (2017) 127:E1–4. doi: 10.1002/lary.26249, PMID: 27577924

[59] Nyunt TPK Mullol J Snidvongs K . Immune response to fungi in diabetic patients with invasive fungal rhinosinusitis. Asian Pac J Allergy Immunol. (2020) 38:233–8. doi: 10.12932/AP-080620-0874, PMID: 33068369

[60] Schell WA . Unusual fungal pathogens in fungal rhinosinusitis. Otolaryngol Clin North Am. (2000) 33:367–73. doi: 10.1016/s0030-6665(00)80011-0, PMID: 10736410

[61] Barbara M Elzayat S Lotfy A Sabino L Bandiera G Elsherif HS . Invasive fungal rhinosinusitis associated with covid-19: Course changes and prognosis predictors. Acta Otorrinolaringol Esp (Engl Ed). (2023) 74:243–52. doi: 10.1016/j.otoeng.2022.11.010, PMID: 36460059

[62] De Boeck I van den Broek MFL Allonsius CN Spacova I Wittouck S Martens K . Lactobacilli have a niche in the human nose. Cell Rep. (2020) 31:107674. doi: 10.1016/j.celrep.2020.107674, PMID: 32460009

[63] Liang Y Xie R Xiong X Hu Z Mao X Wang X . Alterations of nasal microbiome in eosinophilic chronic rhinosinusitis. J Allergy Clin Immunol. (2023) 151:1286–95.e2. doi: 10.1016/j.jaci.2022.11.031, PMID: 36736796

[64] Yuan Y Wang C Wang G Guo X Jiang S Zuo X . Airway microbiome and serum metabolomics analysis identify differential candidate biomarkers in allergic rhinitis. Front Immunol. (2021) 12:771136. doi: 10.3389/fimmu.2021.771136, PMID: 35069544 PMC8766840

[65] He S Chen W Wang X Xie X Liu F Ma X . Deep learning radiomics-based preoperative prediction of recurrence in chronic rhinosinusitis. iScience. (2023) 26:106527. doi: 10.1016/j.isci.2023.106527, PMID: 37123223 PMC10139989

[66] Meng Y Zhang L Lou H Wang C . Predictive value of computed tomography in the recurrence of chronic rhinosinusitis with nasal polyps. Int Forum Allergy Rhinol. (2019) 9:1236–43. doi: 10.1002/alr.22355, PMID: 31237991

[67] Zhu KZ He C Li Z Wang PJ Wen SX Wen KX . Development and multicenter validation of a novel radiomics-based model for identifying eosinophilic chronic rhinosinusitis with nasal polyps. Rhinology. (2023) 61:132–43. doi: 10.4193/Rhin22.361, PMID: 36602548

[68] Idowu OO Soderlund KA Laguna B Ashraf DC Arnold BF Grob SR . Magnetic resonance imaging prognostic findings for visual and mortality outcomes in acute invasive fungal rhinosinusitis. Ophthalmology. (2022) 129:1313–22. doi: 10.1016/j.ophtha.2022.06.020, PMID: 35768053

[69] Gode S Turhal G Ozturk K Aysel A Midilli R Karci B . Acute invasive fungal rhinosinusitis: Survival analysis and the prognostic indicators. Am J Rhinol Allergy. (2015) 29:e164–9. doi: 10.2500/ajra.2015.29.4245, PMID: 26637563

[70] Choi YR Kim J-H Min HS Won J-K Kim HJ Yoo R-E . Acute invasive fungal rhinosinusitis: Mr imaging features and their impact on prognosis. Neuroradiology. (2018) 60:715–23. doi: 10.1007/s00234-018-2034-0, PMID: 29774383

[71] Patel VA LePhong CD Osterbauer B Gomez G Don DM Ference EH . Pediatric invasive fungal rhinosinusitis: A comprehensive analysis of prognostic factors for survival. Laryngoscope. (2022) 133:1239–50. doi: 10.1002/lary.30310, PMID: 35876111

[72] Cho H-J Jang M-S Hong SD Chung S-K Kim HY Dhong H-J . Prognostic factors for survival in patients with acute invasive fungal rhinosinusitis. Am J Rhinol Allergy. (2015) 29:48–53. doi: 10.2500/ajra.2015.29.4115, PMID: 25590320

[73] Wang J Shen S Yan B He Y Zhang G Shan C . Individual exposure of ambient particulate matters and eosinophilic chronic rhinosinusitis with nasal polyps: Dose-response, mediation effects and recurrence prediction. Environ Int. (2023) 177:108031. doi: 10.1016/j.envint.2023.108031, PMID: 37327504

[74] Bousquet J Shamji MH Anto JM Schunemann HJ Canonica GW Jutel M . Patient-centered digital biomarkers for allergic respiratory diseases and asthma: The aria-eaaci approach - aria-eaaci task force report. Allergy. (2023) 78:1758–76. doi: 10.1111/all.15740, PMID: 37042071

[75] Wu Q Chen J Ren Y Qiu H Yuan L Deng H . Artificial intelligence for cellular phenotyping diagnosis of nasal polyps by whole-slide imaging. EBioMedicine. (2021) 66:103336. doi: 10.1016/j.ebiom.2021.103336, PMID: 33857906 PMC8050855

[76] Pitlick MM Li JT Pongdee T . Current and emerging biologic therapies targeting eosinophilic disorders. World Allergy Organ J. (2022) 15:100676. doi: 10.1016/j.waojou.2022.100676, PMID: 35983569 PMC9356173

[77] Okano M Kanai K Oka A . Pathogenesis-based application of biologics for chronic rhinosinusitis: Current and future perspectives. Auris Nasus Larynx. (2024) 51:371–8. doi: 10.1016/j.anl.2023.08.005, PMID: 37743131

[78] Lipworth BJ Chan R . The choice of biologics in patients with severe chronic rhinosinusitis with nasal polyps. J Allergy Clin Immunol Pract. (2021) 9:4235–8. doi: 10.1016/j.jaip.2021.07.023, PMID: 34332173

[79] Jansen F Becker B Eden JK Breda PC Hot A Oqueka T . Dupilumab (dupixent((r))) tends to be an effective therapy for uncontrolled severe chronic rhinosinusitis with nasal polyps: Real data of a single-centered, retrospective single-arm longitudinal study from a university hospital in Germany. Eur Arch Otorhinolaryngol. (2023) 280:1741–55. doi: 10.1007/s00405-022-07679-y, PMID: 36242612 PMC9988751

[80] De Schryver E Derycke L Calus L Holtappels G Hellings PW Van Zele T . The effect of systemic treatments on periostin expression reflects their interference with the eosinophilic inflammation in chronic rhinosinusitis with nasal polyps. Rhinology. (2017) 55:152–60. doi: 10.4193/Rhino16.314, PMID: 28501884

[81] Gevaert P Lang-Loidolt D Lackner A Stammberger H Staudinger H Van Zele T . Nasal il-5 levels determine the response to anti-il-5 treatment in patients with nasal polyps. J Allergy Clin Immunol. (2006) 118:1133–41. doi: 10.1016/j.jaci.2006.05.031, PMID: 17088140

[82] Safia A Khater A Abd Elhadi U Merchavy S Karam M . Optimizing biologic treatment selection in chronic rhinosinusitis with nasal polyps: A network meta-analysis of efficacy and safety across 22 rcts. Pharm (Basel). (2025) 18:1455. doi: 10.3390/ph18101455, PMID: 41155574 PMC12566755

[83] Buchheit KM Lewis E Gakpo D Hacker J Sohail A Taliaferro F . Mepolizumab targets multiple immune cells in aspirin-exacerbated respiratory disease. J Allergy Clin Immunol. (2021) 148:574–84. doi: 10.1016/j.jaci.2021.05.043, PMID: 34144111 PMC9096876

[84] Bachert C Sousa AR Lund VJ Scadding GK Gevaert P Nasser S . Reduced need for surgery in severe nasal polyposis with mepolizumab: Randomized trial. J Allergy Clin Immunol. (2017) 140:1024–31 e14. doi: 10.1016/j.jaci.2017.05.044, PMID: 28687232

[85] Han JK Bachert C Fokkens W Desrosiers M Wagenmann M Lee SE . Mepolizumab for chronic rhinosinusitis with nasal polyps (synapse): A randomised, double-blind, placebo-controlled, phase 3 trial. Lancet Respir Med. (2021) 9:1141–53. doi: 10.1016/S2213-2600(21)00097-7, PMID: 33872587

[86] Cavaliere C Segatto M Ciofalo A Colizza A Minni A Messineo D . Benralizumab reduces eosinophils and inflammatory markers in patients with severe eosinophilic asthma and chronic rhinosinusitis with nasal polyps: A pilot real-life study. Immunol Lett. (2022) 248:70–7. doi: 10.1016/j.imlet.2022.06.009, PMID: 35752279

[87] Bachert C Han JK Desrosiers MY Gevaert P Heffler E Hopkins C . Efficacy and safety of benralizumab in chronic rhinosinusitis with nasal polyps: A randomized, placebo-controlled trial. J Allergy Clin Immunol. (2022) 149:1309–17 e12. doi: 10.1016/j.jaci.2021.08.030, PMID: 34599979

[88] Gevaert P Omachi TA Corren J Mullol J Han J Lee SE . Efficacy and safety of omalizumab in nasal polyposis: 2 randomized phase 3 trials. J Allergy Clin Immunol. (2020) 146:595–605. doi: 10.1016/j.jaci.2020.05.032, PMID: 32524991

[89] Bidder T Sahota J Rennie C Lund VJ Robinson DS Kariyawasam HH . Omalizumab treats chronic rhinosinusitis with nasal polyps and asthma together-a real life study. Rhinology. (2018) 56:42–5. doi: 10.4193/Rhino17.139, PMID: 29288573

[90] Zhang YY Zhang M Zhang JQ Li QQ Lu MP Cheng L . Combination of omalizumab with allergen immunotherapy versus immunotherapy alone for allergic diseases: A meta-analysis of randomized controlled trials. Int Forum Allergy Rhinol. (2024) 14:794–806. doi: 10.1002/alr.23268, PMID: 37715592

[91] Yu C Wang K Cui X Lu L Dong J Wang M . Clinical efficacy and safety of omalizumab in the treatment of allergic rhinitis: A systematic review and meta-analysis of randomized clinical trials. Am J Rhinol Allergy. (2020) 34:196–208. doi: 10.1177/1945892419884774, PMID: 31672020

[92] Bai J Tan BK . B lineage cells and ige in allergic rhinitis and crswnp and the role of omalizumab treatment. Am J Rhinol Allergy. (2023) 37:182–92. doi: 10.1177/19458924221147770, PMID: 36848269 PMC10830379

[93] Fujieda S Matsune S Takeno S Ohta N Asako M Bachert C . Dupilumab efficacy in chronic rhinosinusitis with nasal polyps from sinus-52 is unaffected by eosinophilic status. Allergy. (2022) 77:186–96. doi: 10.1111/all.14906, PMID: 33993501 PMC9290136

[94] Peters AT Wagenmann M Bernstein JA Khan AH Nash S Jacob-Nara JA . Dupilumab efficacy in patients with chronic rhinosinusitis with nasal polyps with and without allergic rhinitis. Allergy Asthma Proc. (2023) 44:265–74. doi: 10.2500/aap.2023.44.230015, PMID: 37480206

[95] Suzaki I Maruyama Y Kamimura S Hirano K Nunomura S Izuhara K . Residual nasal polyp tissue following dupilumab therapy is associated with periostin-associated fibrosis. Eur Arch Otorhinolaryngol. (2024) 281:1807–17. doi: 10.1007/s00405-023-08336-8, PMID: 37979011

[96] Bachert C Hellings PW Mullol J Naclerio RM Chao J Amin N . Dupilumab improves patient-reported outcomes in patients with chronic rhinosinusitis with nasal polyps and comorbid asthma. J Allergy Clin Immunol Pract. (2019) 7:2447–9.e2. doi: 10.1016/j.jaip.2019.03.023, PMID: 30928658

[97] Weinstein SF Katial R Jayawardena S Pirozzi G Staudinger H Eckert L . Efficacy and safety of dupilumab in perennial allergic rhinitis and comorbid asthma. J Allergy Clin Immunol. (2018) 142:171–7 e1. doi: 10.1016/j.jaci.2017.11.051, PMID: 29355679

[98] Jonstam K Swanson BN Mannent LP Cardell LO Tian N Wang Y . Dupilumab reduces local type 2 pro-inflammatory biomarkers in chronic rhinosinusitis with nasal polyposis. Allergy. (2019) 74:743–52. doi: 10.1111/all.13685, PMID: 30488542 PMC6590149

[99] Tversky J Lane AP Azar A . Benralizumab effect on severe chronic rhinosinusitis with nasal polyps (crswnp): A randomized double-blind placebo-controlled trial. Clin Exp Allergy. (2021) 51:836–44. doi: 10.1111/cea.13852, PMID: 33595845

[100] Hopkins C Han JK Fokkens W Wagenmann M Guyot P Khan AH . Dupilumab versus mepolizumab for chronic rhinosinusitis with nasal polyposis: An indirect treatment comparison. J Allergy Clin Immunol Pract. (2024) 12:3393–401.e15. doi: 10.1016/j.jaip.2024.09.015, PMID: 39326524

[101] Corren J Larson D Altman MC Segnitz RM Avila PC Greenberger PA . Effects of combination treatment with tezepelumab and allergen immunotherapy on nasal responses to allergen: A randomized controlled trial. J Allergy Clin Immunol. (2023) 151:192–201. doi: 10.1016/j.jaci.2022.08.029, PMID: 36223848 PMC12205947

[102] Shen S Yan B Wang M Wu D Wang C Zhang L . Anti-il-4ralpha monoclonal antibody (cm310) in patients with chronic rhinosinusitis with nasal polyps (crowns-2): Rationale and design of a multicenter, randomized, double-blind, placebo-controlled, parallel-group study. Asia Pac Allergy. (2024) 14:118–23. doi: 10.5415/apallergy.0000000000000156, PMID: 39220573 PMC11365676

[103] Drube S Strotmann B Wegner P Jager UM Kuchler C Andreas N . Interleukin-3 stabilizes cd124/il-4alpha surface expression in mast cells via tyk2 and stat6. Immunology. (2023) 169:102–12. doi: 10.1111/imm.13614, PMID: 36440951

[104] Wu J Jones N Chao PH Chan V Hohenwarter L Wu A . Intranasal delivery of low-dose anti-cd124 antibody enhances treatment of chronic rhinosinusitis with nasal polyps. Biomaterials. (2024) 308:122567. doi: 10.1016/j.biomaterials.2024.122567, PMID: 38603825

[105] Fokkens WJ Lund VJ Hopkins C Hellings PW Kern R Reitsma S . Executive summary of epos 2020 including integrated care pathways. Rhinology. (2020) 58:82–111. doi: 10.4193/Rhin20.601, PMID: 32226949

[106] Standylo A Obuchowska A Horaczynska-Wojtas A Mielnik-Niedzielska G . Effects of probiotic supplementation during chronic rhinosinusitis on the microbiome. J Clin Med. (2024) 13:1726. doi: 10.3390/jcm13061726, PMID: 38541951 PMC10971434

[107] Abreu NA Nagalingam NA Song Y Roediger FC Pletcher SD Goldberg AN . Sinus microbiome diversity depletion and corynebacterium tuberculostearicum enrichment mediates rhinosinusitis. Sci Transl Med. (2012) 4:151ra124. doi: 10.1126/scitranslmed.3003783, PMID: 22972842 PMC4786373

[108] Wise SK Damask C Roland LT Ebert C Levy JM Lin S . International consensus statement on allergy and rhinology: Allergic rhinitis - 2023. Int Forum Allergy Rhinol. (2023) 13:293–859. doi: 10.1002/alr.23090, PMID: 36878860

[109] Patterson TF Thompson GR 3rd Denning DW Fishman JA Hadley S Herbrecht R . Executive summary: Practice guidelines for the diagnosis and management of aspergillosis: 2016 update by the infectious diseases society of america. Clin Infect Dis. (2016) 63:433–42. doi: 10.1093/cid/ciw444, PMID: 27481947 PMC4967611

[110] Song J Wang M Wang C Zhang L . Olfactory dysfunction in chronic rhinosinusitis: Insights into the underlying mechanisms and treatments. Expert Rev Clin Immunol. (2023) 19:993–1004. doi: 10.1080/1744666X.2023.2235891, PMID: 37432663

[111] Ma Y Yang XH Tu Y Athari SS . Survey the effect of drug treatment on modulation of cytokines gene expression in allergic rhinitis. Fundam Clin Pharmacol. (2023) 37:340–6. doi: 10.1111/fcp.12847, PMID: 36314138

[112] Bernstein JA Bernstein JS Makol R Ward S . Allergic rhinitis: A review. JAMA. (2024) 331:866–77. doi: 10.1001/jama.2024.0530, PMID: 38470381

[113] Yang H Mao H Wang F Guo Q Chu J Zhao X . Clinical efficacy and safety study of loratadine combined with glucocorticoid nasal spray in the treatment of pediatric bronchial asthma with seasonal allergic rhinitis. J Asthma. (2024) 61:1–8. doi: 10.1080/02770903.2024.2379410, PMID: 39007891

[114] Duman Senol H Topyildiz E Ekici B Gulen F Demir E . Effectiveness and adverse reactions to subcutaneous immunotherapy in children with allergic rhinitis/asthma. Int J Pediatr Otorhinolaryngol. (2022) 162:111292. doi: 10.1016/j.ijporl.2022.111292, PMID: 36007303

[115] Demoly P Molimard M Bergmann JF Delaisi B Gouverneur A Vadel J . Impact of liquid sublingual immunotherapy on asthma onset and progression in patients with allergic rhinitis: A nationwide population-based study (efficapsi study). Lancet Reg Health Eur. (2024) 41:100915. doi: 10.1016/j.lanepe.2024.100915, PMID: 38707866 PMC11066575

[116] Jiang S Xie S Tang Q Zhang H Xie Z Zhang J . Evaluation of intralymphatic immunotherapy in allergic rhinitis patients: A systematic review and meta-analysis. Mediators Inflamm. (2023) 2023:9377518. doi: 10.1155/2023/9377518, PMID: 37197570 PMC10185423

[117] Sawane K Nagatake T Hosomi K Hirata SI Adachi J Abe Y . Dietary omega-3 fatty acid dampens allergic rhinitis via eosinophilic production of the anti-allergic lipid mediator 15-hydroxyeicosapentaenoic acid in mice. Nutrients. (2019) 11:2868. doi: 10.3390/nu11122868, PMID: 31766714 PMC6950470

[118] Ren M Wang Y Lin L Li S Ma Q . Alpha-linolenic acid screened by molecular docking attenuates inflammation by regulating th1/th2 imbalance in ovalbumin-induced mice of allergic rhinitis. Molecules. (2022) 27:5893. doi: 10.3390/molecules27185893, PMID: 36144628 PMC9501164

[119] Chen Z Xu Q Liu Y Wei Y He S Lin W . Vancomycin-induced gut microbiota dysbiosis aggravates allergic rhinitis in mice by altered short-chain fatty acids. Front Microbiol. (2022) 13:1002084. doi: 10.3389/fmicb.2022.1002084, PMID: 36439824 PMC9687373

[120] Xiaodong X Tao L Jianmin L Jing Z Bing Z Jintao D . Crocin inhibits the type 2 inflammatory response produced by ilc2s in eosinophilic nasal polyps. Am J Rhinol Allergy. (2023) 37:656–69. doi: 10.1177/19458924231185296, PMID: 37424236

[121] Zhang JJ He XC Zhou M Liu QD Xu WZ Yan YJ . Xiao-qing-long-tang ameliorates ova-induced allergic rhinitis by inhibiting ilc2s through the il-33/st2 and jak/stat pathways. Phytomedicine. (2023) 119:155012. doi: 10.1016/j.phymed.2023.155012, PMID: 37586158

[122] Nazari J Yadegari N Khodam S Almasi-Hashian A Amini S . Effect of consumption of whole-wheat breads on fbs, hba1c, and blood lipids in patients with type 2 diabetes. Prev Nutr Food Sci. (2021) 26:269–74. doi: 10.3746/pnf.2021.26.3.269, PMID: 34737987 PMC8531422

